# Increased prevalence of intellectual disabilities in higher-intensity mental healthcare settings

**DOI:** 10.1192/bjo.2021.28

**Published:** 2021-04-22

**Authors:** Jeanet G. Nieuwenhuis, Peter Lepping, Niels L. Mulder, Henk L. I. Nijman, Mike Veereschild, Eric O. Noorthoorn

**Affiliations:** Department of VGGNet, GGNet, the Netherlands; Centre for Mental Health and Society, Betsi Cadwaladr University, Wales, UK; Department of Psychiatry, Erasmus University Rotterdam, the Netherlands; Behavioural Science Institute, Radboud University, the Netherlands; Department of VGGNet, GGNet, the Netherlands; Department of Research and Development, GGNet, the Netherlands

**Keywords:** Intellectual disability, community mental health teams, out-patient treatment, SCIL, in-patient treatment

## Abstract

**Background:**

It has been suggested that people with intellectual disabilities have a higher likelihood to develop psychiatric disorders, and that their treatment prognosis is relatively poor.

**Aims:**

We aimed to establish the prevalence of intellectual disability in different mental healthcare settings, and estimate percentage of cognitive decline. We hypothesised that the prevalence of intellectual disabilities increases with intensity of care.

**Method:**

A cross-sectional study was conducted in different settings in a mental healthcare trust in the Netherlands. We used the Screener for Intelligence and Learning Disabilities (SCIL) to identify suspected mild intellectual disability (MID) or borderline intellectual functioning (BIF). We identified patients with a high level of education and low SCIL score to estimate which patients may have had cognitive decline.

**Results:**

We included 1213 consecutive patients. Over all settings, 41.4% of participating patients were positive for MID/BIF and 20.2% were positive for MID only. Prevalence of suspected MID/BIF increased by setting, from 27.1% in out-patient settings to 41.9% in flexible assertive community treatment teams and admission wards, to 66.9% in long-stay wards. Only 85 (7.1%) of all patients were identified as possibly having cognitive decline. Of these, 25.9% were in long-stay wards and had a diagnosis of schizophrenia or substance use disorder.

**Conclusions:**

Low intellectual functioning is common in Dutch mental healthcare settings. Only a modest number of patients were identified as suffering from cognitive decline rather than suspected MID/BIF from birth. Therefore, we recommend improved screening of psychiatric patients for intellectual functioning at the start of treatment.

## Prevalence of intellectual disabilty in mental health care

In the Netherlands, as in many other European countries, care and treatment of people with intellectual disability and psychiatric problems became separated from psychiatric care for those without intellectual disability in the 1950s and 1960s. Institutions for patients with intellectual disability and general mental health were separately commissioned by different funding streams in the Netherlands, and since then, each institution's knowledge of the other has diminished over the years. As we know from two previous studies,^1–3^ the prevalence of mild intellectual disability (MID) or borderline intellectual functioning (BIF) is much higher in (general) mental healthcare, as may be expected from the prevalence estimations in the general population. This finding was remarkable, given the background of the development of separate intellectual disability services alongside standard psychiatric care in the Netherlands. In the study, more than 40% of psychiatric in-patients and out-patients screened were suspected to have MID/BIF, using the Screener for Intelligence and Learning Disability (SCIL).^4^ The study validated the SCIL against the Wechsler Adult Intelligence Scale (WAIS)^5^ in participating patients with severe mental illness (SMI). In the Netherlands, the SCIL is a widely accepted screener used in psychiatric and forensic settings.

The population prevalence of MID is estimated to be 0.7 ± 1.3% in Western countries.^6,7^ On the basis of the normal distribution of intelligence in the general population, 2.1% would have an IQ in the 50–70 range (MID) and 13.6% would have an IQ in the 71–84 range (BIF). Possibly, persons with MID/BIF have a higher likelihood of requiring psychiatric care, and relatively often this need for care may be long term and intense.

## Improper diagnosis may lead to developing SMI

For a number of decades, there is an awareness in psychiatry that patients with schizophrenia, substance and alcohol use disorders and bipolar disorder are at risk of developing cognitive decline,^8,9^ and so in the assessment of intellectual impairment, cognitive decline needs to be ruled out. To our knowledge, there are no studies examining the prevalence of MID/BIF in general psychiatry, correcting for possible impaired cognitive functioning, either at birth or acquired in childhood or after 18 years of age.

## Aim of this study

This study investigated intellectual disability and its possible association with cognitive decline in different general mental healthcare settings, each providing an increasingly longer-term treatment. When MID/BIF is not properly identified by clinicians, this may lead to missed, improper or false diagnosis and treatment,^10^ followed by a longer history of psychiatric care, lower quality of life, worse functioning and possible higher care costs.^11^ As such, these patients may develop SMI.^12^ Patients with SMI may be defined as having one or more psychiatric disorders (psychosis, severe depression, personality disorders and bipolar disorder, perhaps in combination with several other disorders), together with social functioning problems for at least 2 years.^13^ Clinical treatment of schizophrenia, mood disorders and personality disorders is different when a patient has an intellectual disability.^14^ Treatment for addiction is also different in a number of aspects if an intellectual disability has to be taken into account.^15,16^ A clear diagnosis at an early stage is therefore important in preventing long-term care dependency.

The aim of this study was to investigate the prevalence of a possible MID/BIF and possible cognitive decline in different mental healthcare settings in the Netherlands. In addition, we investigated the association between MID/BIF and patient characteristics such as age, gender, diagnosis and global functioning. Our hypothesis was that lower intellectual functioning is associated with a higher prevalence of SMI, a more chronic disease course, higher care intensity and worse functioning.

## Method

The study was conducted and reported in accordance with the Strengthening the Reporting of Observational Studies in Epidemiology guidelines for reporting observational studies.^17^ Screening of potential intellectual disabilities was done from May 2014 to January 2019. All patients at participating wards or care centres were asked to join the study. We used consecutive sampling, thus asking all new patients to participate; all participants provided informed consent. This allowed for non-response analysis, adding to the clinical validity of the findings.^18^

### Setting

We collected a consecutive sample of patients treated with four different types of care in a mental healthcare trust in the east of the Netherlands, covering a catchment area of 630 000 inhabitants. This Trust covers all specialised mental healthcare in the catchment area, with an annual total of about 18 000 out-patient referrals and approximately 2500 in-patient referrals, of which approximately 200 patients reside in long-stay wards. It is a standard mental health trust, of which the Netherlands has 24. The four types of care included were:

- Out-patient clinics, where patients were referred to after having been treated with insufficient effect by a general practitioner, community nurse or psychologist.
- Flexible assertive community treatment (FACT) teams, specialising in daily (out-patient) support and treatment for patients with SMI. In the Netherlands, FACT teams are multidisciplinary out-patient teams with between eight and ten professionals, such as a psychiatrist, a psychologist, several nurses and social workers, in general taking care of 200 patients with SMI.^19^
- General admission wards, admitting both first-onset patients and patients referred from FACT teams or out-patient clinics. In addition, patients at these wards were eligible for the study after at least 6 days on the ward.
- Long-stay wards, providing residential care for patients with SMI. The teams at these wards have a similar setup to the FACT teams. Patients all have a long history of receiving professional support and treatment, primarily in the FACT teams.

Patients were excluded if they had an inadequate grasp of the Dutch language; lack of cooperation; or an inability, in the assessor's opinion, to concentrate for at least 20 min to engage in the test as outlined in the instruction.^20^

### Measures

#### MID/BIF screening with the SCIL

We used the SCIL to detect patients suspected for MID or BIF.^20^ The SCIL was first used in several published studies in forensic psychiatry in the Netherlands.^21^ Translation for use in English is in preparation. The SCIL comprises 14 questions, including education level and small tasks that are intended to provide overall insight into a patient's cognitive abilities.^20^ It was developed specifically to detect MID/BIF (IQ 50–85) in people in a range of settings, such as healthcare or social service settings, police stations and shelters for the homeless. The SCIL adds to other screeners for intellectual disability, such as the Hayes Ability Screening Index,^22^ because it screens for BIF in addition to MID.

The SCIL was validated in an adult sample by comparing the scores obtained with test results from the WAIS III. The reliability of the SCIL, as expressed in Cronbach's alpha, was good (0.83 in 318 adults). The area under the curve value was 0.93, which is excellent. With ≤19 as a cut-off score, the SCIL accurately classified 82% of people with MID/BIF. Of the ten people without MID/BIF, nine (89%) were classified correctly as having no MID/BIF. In accordance with the SCIL manual, administering the SCIL requires no specific clinical skills.

Recently, the SCIL had been validated in patients with SMI in FACT teams.^3^ The Cronbach's alpha of the SCIL in that sample was 0.73. The area under the curve value was 0.81 for detecting MID/BIF and 0.81 for detecting MID, with percentages of correctly classified individuals of 73% and 79%, respectively. We used two cut-off scores, 19 and 15. Scoring >19 implied no MID/BIF, and scoring ≤19 implied suspected MID/BIF. The cut-off point of ≤15 implies an MID.^20^ In the following descriptions, we use two cut-off points: 19 for MID or BIF. and 15 for MID only.

#### Cognitive decline

The SCIL does not distinguish between impaired intellectual functioning caused by cognitive decline and intellectual disability from birth. To detect a potential cognitive decline after 18 years of age, we verified patients’ school reports and qualifications in their medical file. We categorised the school qualifications into four education levels, which are related to estimated IQ (WAIS) levels. For this, we identified the educational attainment of the participants. We categorised >60 different educational data and certificates into four categories. By accessing publicly available information, we estimated and verified the content of the educational data and certificates to WAIS levels. Two team members coded the school certificates and obtained consensus in a final listing.

Education level 4 corresponds to an estimated IQ outcome on the WAIS of ≥120, level 3 corresponds to an IQ of 110–120, level 2 corresponds to an IQ of 85–110 and level 1 corresponds to an estimated IQ of 50–85. We compared these levels with SCIL outcomes. An education level of 2, 3 and 4 with a current SCIL of ≤19, implying low intellectual functioning after a reasonable educational attainment, may suggest cognitive decline. Patient characteristics of patients with a possible cognitive decline were compared with the patient characteristics of all other patients in the sample. We performed this comparison to understand whether the patient characteristics associated with intellectual disability were the same as those associated with cognitive decline.

#### Demographic and medical information

The following information was extracted from digital medical notes: age, gender, psychiatric diagnosis (DSM-IV-TR, as assessed by the psychiatrist) and Global Assessment of Functioning (GAF) score. In all samples, we included retrospective file information from the 5 years before the SCIL was conducted. A maximum of four primary DSM diagnoses were included.

### Statistical analyses

We calculated the odds ratios when comparing groups, to understand the extent of differences between groups. Where appropriate, differences between groups were tested by means of *t*-tests or *χ*^2^-tests. An alpha of 0.001 was used, because of the large numbers in the study. Missing values were recorded and reported where they may be expected to have an effect on the findings.

### Ethics

Ethical approval for the study was provided in 2014 by the ethical board of the University of Twente, Enschede, the Netherlands. All procedures performed in the current study were in accordance with the Helsinki Declaration of 1975, as revised in 2008, and with comparable ethical standards. Data were analysed on the basis of fully anonymised data that allowed none of the cases to be traced to an individual.

## Results

### Patients

We asked 1616 consecutive patients to participate; there was an available SCIL score in 1213 cases (75.1%). The response did not vary greatly across settings. At the out-patient clinics the response rate was 71.3%, followed by the FACT teams with 72.9%. At the long-stay wards the response rate was 75.8%, whereas at the general admission wards it was 79.2%. We included 313 patients from out-patient services, 291 patients from FACT teams, 452 patients from admission wards and 157 patients from long-stay wards.

Patients in the out-patient services were significantly younger than in the admission wards, the FACT teams and the long-stay wards. The long-stay wards had admitted more male patients.

### SCIL scores across the settings

Table 1 presents the distribution of SCIL categories across the four examined settings. The results show that overall, 41.4% of the 1213 included patients had an SCIL score of ≤19 (corresponding to suspected MID/BIF) and 20.2% had an SCIL score of ≤15 (corresponding to likely MID). Of the 313 general out-patients interviewed, 27.2% had an SCIL score of ≤19 (suspected MID/BIF), and 10.2% had an SCIL score of ≤15 (MID). The 291 patients interviewed at FACT teams showed a significantly higher prevalence of suspected intellectual disabilities; 41.2% of the FACT team patients had an SCIL score of ≤19 and 20.6% had an SCIL score of ≤15. Of the 452 patients interviewed at regular admission wards, 42.5% had an SCIL score of ≤19 (suspected MID/ BIF) and 19.0% had an SCIL score of ≤15 (suspected MID). The 157 patients at the long-stay ward had the highest prevalence of positive SCIL scores; 66.9% had an SCIL score of ≤19 and 42.7% had an SCIL score of ≤15. This increase is also reflected in differences in odds ratios over the four settings, with the out-patient services at the lower end (SCIL ≤ 19 odds ratio 0.43, SCIL ≤ 15 odds ratio 0.37) and the long-stay wards at the higher end (SCIL ≤ 19 odds ratio 3.35, SCIL ≤ 15 odds ratio 3.67).

**Table 1:** Distribution of MID/BIF as identified by SCIL scores and patient characteristics across settings

	Total	Out-patient services	Admission wards	FACT teams	Long-stay in-patient wards	*P-*value
*n*	1616	439	571	399	207	
Response	1213 (75.1%)	313 (71.3%)	452 (79.1%)	291 (75.0%)	157 (75.8%)	
Mean age (s.d.)	43.1 (11.8)	39.3 (10.8)	43.9 (12.3)	46.5 (10.9)	45.3 (13.3)	<0.001
Gender						
Male	790 (48.9%)	200 (45.9%)	270 (47.3%)	177 (44.4%)	143 (69.1%)	<0.001
Female	826 (51.1%)	239 (54.4%)	301 (52.7%)	222 (55.6%)	64 (30.9%)	
Education level						
Low	823 (61.7%)	227 (59.4%)	276 (60.5%)	198 (60.4%)	122 (72.6%)	0.020
High	511 (31.6%)	155 (40.6%)	180 (39.5%)	130 (39.6%)	46 (27.4%)	
Not assessed	282 (17.4%)	57 (12.9%)	115 (20.1%)	71 (17.8%)	39 (18.9%)	<0.001
SCIL outcome						
% No SCIL	403 (24.9%)	126 (28.7%)	119 (20.8%)	108 (27.1%)	50 (24.2%)	0.024
Intellectual functioning						
% SCIL negative (>19)	711 (58.6%)	228 (72.8%)	260 (57.5%)	171 (58.8%)	52 (33.1%)	<0.001
% SCIL positive (≤19)	502 (41.4%)	85 (27.2%)	192 (42.5%)	120 (41.2%)	105 (66.9%)	
BIF SCIL positive/negative	Odds ratio	0.43	1.07	0.99	3.35	
	95% CI	0.33–0.57	0.85–1.36	0.76–1.30	2.35–4.78	
	*P*-value	<0.001	0.551	0.505	<0.001	
Mild intellectual disability						
% SCIL >15	968 (79.8%)	281 (89.8%)	366 (81.0%)	231 (79.4%)	90 (57.3%)	<0.001
% SCIL ≤15	245 (20.2%)	32 (10.2%)	86 (19.0%)	60 (20.6%)	67 (42.7%)	
SCIL ≤15/SCIL >15	Odds ratio	0.37	0.89	1.03	3.67	
	95% CI	0.25–0.55	0.66–1.19	0.75–1.43	2.57–5.24	
	*P*-value	<0.001	0.240	0.448	<0.001	
Cognitive decline						
High education level with high SCIL	1128 (93.0%)	303 (96.8%)	420 (92.9%)	270 (92.8%)	135 (86.0%)	<0.001
High education level with low SCIL	85 (7.1%)	10 (3.2%)	32 (7.1%)	21 (7.2%)	22 (14%)	
SCIL in line with education level/SCIL not in line with education level	Odds ratio	0.36	1.02	1.04	2.57	
	95% CI	0.18–0.71	0.65–1.60	0.63–1.74	1.53–4.31	
	*P-*value	<0.001	0.513	0.481	<0.001	

MID, mild intellectual disability; BIF, borderline intellectual functioning; SCIL, Screener for Intelligence and Learning Disabilities; FACT, flexible assertive community treatment team.

### Diagnosis

When we investigate the differences in diagnosis between those assessed with the SCIL and those who were not (because they did not want to or could not participate), we observed no significant differences in diagnoses between patients (Table 2).

**Table 2:** Distribution of patient characteristics and diagnosis in patients not assessed with the SCIL, with SCIL scores above and below 19 (borderline intellectual functioning and mild intellectual disability) and with SCIL scores above and below 15 (mild intellectual disability)

	*n*		Below borderline intellectual functioning/mild intellectual disability				Mild intellectual disability			
		% No SCIL	% SCIL negative (>19)	% SCIL positive (≤19)	SCIL positive/SCIL negative		% SCIL >15	% SCIL ≤15	SCIL ≤15/SCIL >15	
					Odds ratio	95% CI			Odds ratio	95% CI
*n*	1616	403	711	502			968	245		
Response	1213 (75.1%)^a^	302 (24.9%)^b^	533 (43.9%)^b^	378 (31.2%)^b^						
Mean age (s.d.)		44.9 (11.7)	42.1 (11.5)	43.1 (12.4)			42.2 (11.6)	43.9 (12.7)		
Gender										
Male	790 (48.9%)	193 (47.9%)	336 (47.3%)	261 (52.0%)	0.83	0.66–1.04	462 (47.7%)	135 (55.1%)	0.74	0.56–0.99
Female	826 (51.1%)	210 (52.1%)	375 (52.7%)	241 (48.0%)			506 (52.3%)	110 (44.9%)		
Education level^c^^,^^d^										
Low	823 (61.7%)	124 (68.9%)	321 (46.5%)	378 (81.6%)	0.20	0.15–0.26	502 (54.4%)	197 (84.9%)	0.21	0.15–0.31
High	511 (38.3%)	56 (31.1%)	370 (53.5%)	85 (18.4%)			420 (46.5%)	35 (15.1%		
Not assessed	282 (17.4%)	122 (30.2%)	20 (2.8%)	39 (7.8%)						
Diagnosis										
Adjustment disorder	63 (3.9%)	11 (2.7%)	31 (4.4%)	21 (4.2%)	0.96	0.55–1.69	42 (4.3%)	10 (4.1%)	0.94	0.47–1.90
Anxiety disorder^c^^,^^d^	239 (14.8)	124 (17.4%)	124 (17.4%)	55 (11.0%)	0.58	0.41–0.82	153 (15.8%)	26 (10.6%)	0.63	0.41–0.99
Depression^c^^,^^d^	481 (29.8%)	127 (31.5%)	252 (35.4%)	102 (20.3%)	0.47	0.36–0.61	311 (32.1%)	43 (17.6%)	0.45	0.32–0.64
PTSD diagnosis	301 (18.6%)	77 (19.1%)	125 (17.6%)	99 (19.7%)	1.15	0.86–1.54	180 (18.6%)	44 (18.0%)	0.96	0.67–1.38
Bipolar disorder	163 (10.1%)	31 (7.7%)	73 (10.3%)	59 (11.8%)	1.16	0.81–1.67	10.9 (11.3%)	23 (9.4%)	0.96	0.88–1.05
Psychotic disorders	266 (16.5%)	65 (16.1%)	111 (15.6%)	90 (17.9%)	1.18	0.87–1.60	148 (15.3%)	53 (21.6%)	1.53	1.07–2.17
Schizophrenia^c^^,^^d^	341 (21.1%)	101 (25.1%)	99 (13.9%)	141 (28.1%)	2.41	1.81–3.22	153 (15.8%)	87 (35.5%)	2.93	2.14–4.01
Developmental disorder	213 (13.2%)	49 (12.2%)	97 (13.6%)	67 (13.3%)	0.97	0.69–1.36	135 (13.9%)	29 (11.8%)	0.83	0.54–1.28
Substance use disorder^c^^,^^d^	227 (14.0%)	54 (13.4%)	79 (11.1%)	94 (18.7%)	1.84	1.33–2.54	124 (12.8%)	49 (20.0%)	1.70	1.18–2.45
Personality disorder^a^^,^^b^	669 (41.4%)	186 (46.2%)	309 (43.5%)	174 (34.7%)	0.69	0.54–0.87	409 (42.3%)	74 (30.2%)	0.59	0.44–0.79
Intellectual disability^c^^,^^d^	136 (8.4%)	35 (8.7%)	19 (2.7%)	82 (16.3%)	7.11	4.25–11.88	49 (5.1%)	52 (21.2%)	5.05	3.32–7.69
GAF score <45^c^^,^^d^^,^^e^	564 (36.3%)	147 (37.5%)	208 (30.9%)	209 (42.9%)	1.68	1.32–2.14	300 (32.6%)	117 (48.8%)	1.97	1.48–2.63
Assessed	1616 (100%)	403 (100%)	711 (100%)	502 (100%)			968 (100%)	245 (100%)		

SCIL, Screener for Intelligence and Learning Disabilities; PTSD, post-traumatic stress disorder; GAF, Global Assessment of Functioning.

a. Column percentage (per cent response in patients over the whole group).

b. Row percentage (distribution of the various SCIL groups over the 1213 respondents).

c. Significant difference between SCIL positive and SCIL negative (borderline intellectual functioning), *P* < 0.001 one-sided *χ*^2^-test.

d. Significant difference between SCIL below 15 and above 15 (mild intellectual disability), *P* < 0.001 one-sided *χ*^2^-test.

e. The GAF scores were not administered in 63 (4.5%) out of the 1616 respondents.

When we investigate the differences between the various SCIL groups we did assess, we found that the diagnoses schizophrenia (odds ratio 2.41, 95% CI 1.81–3.22, *P* < 0.001), substance use disorder (odds ratio 1.84, 95% CI 1.33–2.54, *P* < 0.005) and intellectual disability (odds ratio 7.11, 95% CI 4.25–11.88, *P* < 0.001) were significantly more prevalent in patients with an SCIL score of ≤19. The same diagnoses were also more prevalent in patients with an SCIL score of ≤15 (schizophrenia: odds ratio 2.93, 95% CI 2.14–4.01, *P* < 0.001; substance use disorder: odds ratio 1.70, 95% CI 1.18–2.45, *P* < 0.05; intellectual disability: odds ratio 5.05, 95% CI 3.32–7.69, *P* < 0.05). Patients more frequently had a GAF score <45 if they had an SCIL score of ≤19 (odds ratio 1.68, 95% CI 1.32–2.14, *P* < 0.001) or an SCIL score of ≤15 (odds ratio 1.97, 95% CI 1.48–2.63, *P* < 0.001).

Patients with an SCIL score of ≤19 were significantly less frequently diagnosed with anxiety disorder (odds ratio 0.58, 95% CI 0.41–0.82, *P* < 0.001), depression (odds ratio 0.47, 95% CI 0.36–0.61, *P* < 0.001) and personality disorder (odds ratio 0.69, 95% CI 0.54–0.87, *P* = 0.002). When we compare the above diagnoses in patients with an SCIL score above or below 15, the outcomes were nearly the same (odds ratios of 0.63, 0.45 and 0.59, respectively).

When examining the distribution of diagnoses over the four settings, we observed some diagnoses, such as anxiety disorder and depression, occurring more in out-patient services and admission wards, whereas others, such as psychotic disorders, schizophrenia, substance use disorders or a GAF score <45, occurred more in the FACT teams and long-stay wards (Table 3).

**Table 3:** Distribution of diagnosis over settings

Diagnosis		Out-patient clinics	Admission wards	FACT teams	Long-stay wards	*P-*value
Adjustment disorder	63 (3.9%)	18 (4.1%)	30 (5.3%)	10 (2.5%)	5 (2.4%)	0.106
Anxiety disorder	239 (14.8%)	87 (19.8%)	88 (15.4%)	47 (11.8%)	17 (8.2%)	<0.001
Depression	481 (29.8%)	161 (36.7%)	231 (40.5%)	81 (20.3%)	8 (3.9%)	<0.001
PTSD diagnosis	301 (18.6%)	68 (15.5%)	141 (24.7%)	83 (20.8%)	9 (4.3%)	<0.001
Bipolar disorder	163 (10.1%)	20 (4.6%)	77 (13.5%)	54 (13.5%)	12 (5.8%)	<0.001
Psychotic disorder	266 (16.5%)	13 (3.0%)	131 (22.9%)	70 (17.5%)	52 (25.1%)	<0.001
Schizophrenia	341 (21.1%)	3 (0.7%)	91 (15.9%)	115 (28.8%)	132 (63.8%)	<0.001
Developmental disorder	213 (13.2%)	58 (13.2%)	65 (11.4%)	62 (15.5%)	28 (13.5%)	0.314
Substance use disorder	227 (14.0%)	20 (4.6%)	92 (16.1%)	54 (13.5%)	61 (29.5%)	<0.001
Personality disorder	669 (41.4%)	188 (42.8%)	232 (40.6%)	187 (46.9%)	62 (30.0%)	<0.001
Intellectual disability	136 (8.4%)	5 (1.1%)	47 (8.2%)	45 (11.3%)	39 (18.8%)	<0.001
Low GAF score	564 (36.3%)	56 (14.4%)	257 (45.6%)	123 (31.2%)	128 (62.1%)	<0.001

FACT, flexible assertive community treatment team; PTSD, post-traumatic stress disorder; GAF, Global Assessment of Functioning.

### Cognitive decline

Of the 1213 included patients, only 85 (7.1%) had a high education level (levels 2–4) corresponding with a low SCIL score. In contrast to this, in patients with an SCIL score of ≤19, 81.6% had a low education level (level 1). In patients with an SCIL score of ≤15, as many as 84.9% had a low education level (Table 4). We could not clearly identify the education level of 308 patients (27.3%) without suspected cognitive decline (*n* = 1128). In the patients with possible cognitive decline (a high education level and low SCIL), education levels could not be verified in only four patients (4.7%).

**Table 4:** Diagnostic characteristics of patients with a high education level but low SCIL score (likely cognitive decline rather than intellectual disability from birth)

	Low education level and low SCIL score or high education level and high SCIL score	High education level and low SCIL score	SCIL in line with education level/SCIL not in line with education level		*P-*value
			Odds ratio	95% CI	
*n*	1128	85			
%	92.9%	7.1%			
Education level not verifiable	308 (27.3%)	4 (4.7%)			
Diagnosis					
Adjustment disorder	49 (4.3%)	3 (3.5%)	0.80	0.25–2.64	0.498
Anxiety disorder	168 (14.9%)	11 (12.9%)	0.85	0.44–1.63	0.382
Depression	335 (29.7%)	19 (22.4%)	0.68	0.40–1.15	0.093
PTSD diagnosis	208 (18.4%)	16 (18.8%)	1.02	0.58–1.80	0.512
Bipolar disorder	117 (10.4%)	15 (17.6%)	1.85	1.03–3.34	0.038
Psychotic disorders	188 (16.7%)	13 (15.3%)	0.90	0.49–1.66	0.441
Schizophrenia	214 (19.0%)	26 (30.6%)	1.88	1.16–3.06	0.009
Developmental disorder	156 (13.8%)	8 (9.4%)	0.64	0.31–1.37	0.162
Substance use disorder	153 (13.6%)	20 (23.5%)	1.96	1.15–3.33	0.012
Personality disorder	454 (40.2%)	29 (34.1%)	0.77	0.48–1.22	0.159
Intellectual disability	95 (8.4%)	6 (7.1%)	0.83	0.35–1.94	0.426
GAF score <45	377 (34.9%)	40 (48.8%)	1.77	1.13–2.78	0.009
Out-patient wards	303 (26.9%)	10 (11.8%)	0.36	0.18–0.71	0.001
Admission wards	420 (37.2%)	32 (37.6%)	1.02	0.65–1.60	0.513
FACT teams	270 (23.9%)	21 (24.7%)	1.04	0.62–1.74	0.481
Long-stay in-patient wards	135 (12.0%)	22 (25.9%)	2.57	1.53–4.31	0.001

SCIL, Screener for Intelligence and Learning Disabilities; PTSD, post-traumatic stress disorder; GAF, Global Assessment of Functioning; FACT, flexible assertive community treatment team.

**Fig. 1:**
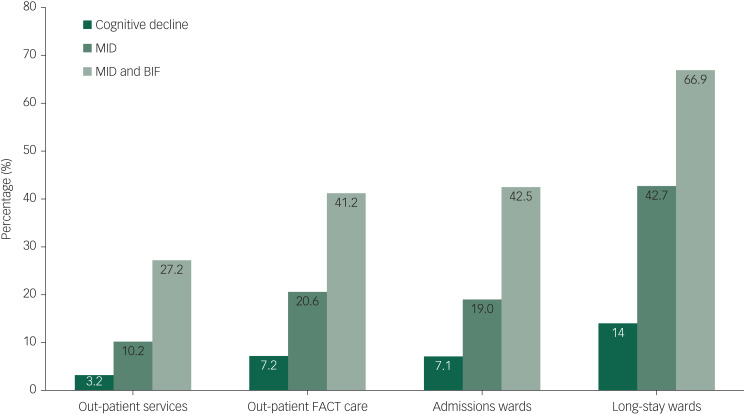
Prevalence of MID, BIF/MID and suspected cognitive decline in the four studied settings of psychiatric care. BIF, borderline intellectual functioning; FACT, flexible assertive community treatment team; MID, mild intellectual disability.

Patients on the long-stay wards were more likely to have a patient history associated with cognitive decline (odds ratio 2.57, *P* < 0.001) than patients from the out-patient services, who were the least likely group to show evidence of cognitive decline (odds ratio 0.36, *P* < 0.001). Figure 1 provides a summary of the proportions of the various SCIL groups (no intellectual disability. suspected BIF and MID) and the proportions of patients with possible cognitive decline over the wards.

### Diagnosis and cognitive decline

In patients with possible cognitive decline, schizophrenia (odds ratio 1.85, 95% CI 1.03–3.34, *P* = 0.009) and a GAF score <45 (odds ratio 1.77, 95% CI 1.13–2.78, *P* = 0.009) were significantly more often associated with the cognitive decline group (Table 4).

## Discussion

Intellectual functioning appears to be a factor that commonly remains unnoticed,^1,2^ but is important in the treatment and recovery of psychiatric patients. We found that a strikingly high number of 41.4% of patients across the four investigated care settings showed a high probability of MID or BIF. This is significantly higher than the prevalence expected in the population, but in keeping with the few existing previous smaller studies.^1–3^ The prevalence increased with the intensity level of the mental healthcare provided (lowest in out-patient settings, highest in long-stay wards). These findings are in line with a recent forensic sample showing prevalence rates as high as 60%.^23^ Importantly, in this study the SCIL findings were validated with concurrent WAIS outcomes. A recently published, retrospective study by Smits et al^24^ showed that patients in FACT teams with possible BIF can benefit more from treatment when professionals know about their lower cognitive level. This same study showed that patients with possible MID, in contrast, did not benefit from a different approach, and hardly recovered.

Considering our current findings, although we did not examine causality, we have to consider the possibility that not recognising intellectual disability in patients at an early stage may lead to poorer treatment outcomes. Only 7.1% of all included patients showed evidence suggesting cognitive decline since adulthood, which is a lower percentage than expected.^5^ Somewhat unsurprisingly, most of these patients were on long-stay wards. A total of 72.6% of patients in the long-stay wards turned out to have a low education level. This suggests that these patients may have already functioned at a lower intellectual level in their youth. However, it cannot be ruled out that current psychotropic medication influences the SCIL outcome. Long-term hospital stay, comorbid mental illness and limited participation in society, especially for the patients at the long-stay in-patient wards, may also limit SCIL outcomes. Nevertheless, the SCIL is an instrument that has no time limit and deliberately assesses early school skills, making it less dependent on current social deprivation or medication effects than the WAIS.

After analysis of the existing evidence, we hypothesised that that lower intellectual functioning is associated with more severe illness, a poorer prognosis and worse functioning. We know from several intellectual disability studies^25,26^ in patients with MID in the UK, the USA and Finland, that schizophrenia, psychotic disorders, aggression and alcohol and substance misuse are often reasons for hospital admission and long-term treatment. Looking at the distribution of diagnoses in the patients with an SCIL score of ≤19, schizophrenia (odds ratio 2.41), substance use disorder (odds ratio 1.84) and intellectual disability (odds ratio 7.11) are significantly more often diagnosed than in patients with an SCIL score of ≥20. In a review article of psychiatric disorders in intellectual disability, Morgan et al^27^ concluded that schizophrenia was overrepresented among patients with additional intellectual disability, especially in those in the borderline and MID range. In addition, Hassiotis et al showed that patients with BIF are at high risk of developing psychotic symptoms.^28^ Our findings are in line with these intellectual disability studies and with more recent studies about schizophrenia in general psychiatry, which showed patients with schizophrenia may have a lower education level because of preadolescent onset of the disease.^29^ A review article from Chapman and Wu^15^ concluded that although the prevalence of alcohol and illicit substance use in the intellectual disability population in the USA is low, the risk of having a substance-related problem is comparatively high. Prevention and treatment programmes for these individuals seem to fail. This emphasises the need to recognise intellectual disability in mental health settings early, to optimise treatment for substance misuse in this patient group. Again, our results are in keeping with these findings. We did not find an increased association of developmental disorders with MID or BIF, despite our substantial sample size. In line with underreporting of intellectual disability, underreporting of developmental disorders cannot be ruled out, as was also shown in a recent study in a long-stay in-patient sample.^30^

In the UK, the National Intellectual Disability Professional Senate defined modern specialist community health services for people with intellectual disability in 2015.^31^ In the Netherlands, this patient group is too often not recognised enough, or MID/BIF may be cited as a reason to exclude such patients from treatment programmes. UK studies have shown that, regardless of the method or model used, increasing knowledge, accessibility and collaboration of both mental health and intellectual disability services improves functioning of patients with intellectual disability and decreases in-patients referrals.^26^ Healthcare providers should develop effective training packages regarding the treatment of intellectual disability in standard mental healthcare settings.

In summary, this study shows that there is a strong association between suspected MID/BIF, diagnoses such schizophrenia and addiction, worse overall functioning and a long history of psychiatric care. The finding that high or low SCIL outcomes are associated with high or low educational attainment level suggests a pre-existing impaired intellectual level. The patient journey usually starts in out-patient services. Professionals’ knowledge of the diagnostic process and treatments, adapted to the cognitive and intellectual needs of patients with BIF and MID, are important for the effectiveness of such treatments. In the Netherlands, the specific needs of patients with intellectual disabilities are often omitted from the training of professionals. We know that psychiatric patients with intellectual disability can significantly benefit from treatment. The literature confirms that patients with BIF/MID living in long-term residential facilities^30^ who are re-diagnosed in a specialised centre for intellectual disability and psychiatry, not only obtained other, but also multiple diagnoses. The interference of the intellectual disability and its interconnection with a lower level of emotional maturity demands a thorough assessment. If not recognised, patients with possibly unidentified BIF and MID may end up being classed as having an SMI, and costs may rise rapidly because of failed treatment approaches. BIF/MID can thus become a significant risk factor for developing chronicity. Therefore, it is important to be aware of the intellectual functioning of each patient. We recommend screening patients for intellectual disability as far as practically possible, as part of any assessment at the start of treatment.

Knowledge about the diagnostic process and effective treatment for patients with BIF and MID are important. We know that treatments are effective for psychiatric patients with intellectual disability. Patients who do not follow the expected path of recovery may benefit from input from intellectual disability specialists, for a diagnostic re-assessment and specialised treatment plan.

A limitation of this study is that cognitive decline remains an estimation within all the patients assessed with the SCIL, and based on education level as documented in the medical file. Both SCIL scores and the categorisation of educational certificates into WAIS levels are estimates. Furthermore, intellectual ability may not necessarily be linked to academic achievements.^32^ In 27% of those patients with an SCIL outcome in line with the SCIL score, and 5% of those with a high education and low SCIL score, the education level could not be verified from the medical file. Also, patients with preadolescent schizophrenia with very early cognitive decline were not detected in this study.^29^ Other factors, such as psychotropic medication, long-term hospital stay, comorbid psychiatric illness and limited participation in society may also have negatively influenced the outcomes of the SCIL.

The strengths of this study are the number of included patients and the high recruitment rate of 75%. To our knowledge, the prevalence of intellectual impairment and cognitive decline of psychiatric patients over different settings has not been studied before. Another strength is the use of the SCIL, as this instrument assesses BIF in addition to MID, adding to current knowledge that primarily focusses on the association of intellectual disability with psychiatric disorders.

In conclusion, this study shows that 40% of patients in a general mental health trust in the Netherlands are suspected for an MID or borderline intellectual disability across different settings, which is far more than expected. Only 7% of those were assessed as having acquired cognitive decline since adolescence. The prevalence of suspected intellectual disability increased in settings providing increasingly more intensive and longer-term treatment. When intellectual disability is not properly identified by clinicians, it may lead to improper or false diagnosis and treatment, poorer functioning and perhaps higher care costs. We therefore recommend that clinicians screen for intellectual functioning at the start of treatment and work together in a multidisciplinary way, to prevent long-term care dependency.

## Data Availability

Data analysis was performed on fully anonymised data, which could in no case be used to identify an individual. Data are available at the Radboud University Nijmegen Repository. The data that support the findings of this study are available on request from the last author, E.N.N. (E.noorthoorn@ggnet.nl). The data are not publicly available due to their containing information that could compromise the privacy of research participants.
